# Hemicrania Continua and Pituitary Microadenoma - Post Hoc Ergo Propter Hoc?: A Case Report With a Side Note on Intra-Sellar Pressure and the Trigemino-Autonomic Reflex

**DOI:** 10.7759/cureus.10223

**Published:** 2020-09-03

**Authors:** Hassan Kesserwani

**Affiliations:** 1 Neurology, Flowers Medical Group, Dothan, USA

**Keywords:** primary headache disorder, nonfunctioning pituitary adenoma

## Abstract

We describe the case of a 38-year-old woman whose headache phenotype transformed from episodic migraine to hemicrania continua (HC) responsive to indomethacin, as expected per diagnostic criteria. Our patient also had a non-functioning pituitary micro-adenoma which is over-represented in the trigeminal autonomic cephalgias (TAC) such as HC, pituitary adenoma being the most common intra-cranial pathology. We explore our case further by outlining in detail the neural supply of the dura of the pituitary fossa, outline the dynamics of intra-sellar pressure (ISP), and posit potential mechanisms of generation of HC in patients with pituitary micro-adenoma. We stress and further explore the remarkable observation that indomethacin, which lowers intracranial pressure, exquisitely resolves the pain of HC. Furthermore, we hypothesize that despite normal ISP, the slight elevation of ISP and mass effect impairs portal venous circulation, which may lead to venous hypertension and/or parasympathetic hyperactivity, which explains the pain and autonomic features of HC.

## Introduction

Hemicrania continua (HC) is a persistent and unremitting unilateral primary headache disorder, lasting at least three months, with intermittent moderate or severe exacerbation. It can be associated with restlessness or aggravation of pain by motion. It has also been associated with at least one of the following autonomic symptoms, mostly parasympathetic: conjunctival injection, lacrimation, nasal congestion, rhinorrhea, eyelid edema, forehead or facial sweating, miosis, mydriasis or ptosis. Other less common symptoms include eye itching, aural and peri-aural swelling, a sense of aural fullness and a swelling of the face and cheek. Migrainous symptoms such as photophobia and phonophobia have also been described, usually occurring during exacerbations [1,2]. HC is differentiated from the trigeminal autonomic cephalgias (TAC) by its temporal characteristics, the former being continuous and the latter episodic. The attacks of cluster headaches usually last minutes to hours and may occur up to eight episodes a day, attacks of paroxysmal hemicrania usually last minutes and their frequency up to 40 episodes a day, and the attacks of short lasting neuralgiform headaches with conjunctival injection (SUNCT) last seconds and may occur up to 200 times a day.

A therapeutic response to indomethacin is an absolute requirement. The nadir dosing threshold is 150 milligrams (mg) daily. Positron emission tomography (PET) scanning has demonstrated abnormal metabolism of the posterior hypothalamus, which is the most proximal parasympathetic center of the brainstem. A study of seven patients prior and post administration of 100 mg intramuscular indomethacin showed there was significant activation of the contralateral posterior hypothalamus and ipsilateral dorsal rostral pons. In addition, there was activation of the ipsilateral ventrolateral midbrain, which extended over the red nucleus and the substantia nigra, and bilateral ponto-medullary junction [3].

Whereas HC is mostly primary, secondary causes such as head trauma, post craniotomy head pain and pituitary adenomas have been associated with HC, in this order of frequency [4]. Pituitary adenoma is the most common intracranial pathology associated with HC [5].

A 38-year-old woman presented with episodic migraine, responding to a calcitonin G related peptide (CGRP) antagonist, which subsequently transformed to HC. She fulfilled the diagnostic criteria of HC and responded to the minimum effective dose of indomethacin, a sine qua non. Our case had an incidental non-functioning micro-adenoma of the pituitary, an intracranial pathology known not to be a stochastic association, but may be causative in the pathogenesis of HC. In the Discussion section, we discussed the neural supply of the pituitary fossa dura, intra-sellar pressure (ISP) and the potential mechanisms of generation of HC in general and specifically in association with pituitary adenomas. In particular, we will focus on the role of the trigeminal autonomic pathway.

## Case presentation

Our patient's headaches began two years prior to presentation with episodic holocranial throbbing headaches lasting six hours. These headaches were triggered by heat and exercise. They were throbbing and associated with photophobia and phonophobia. She averaged four headaches per month. She was diagnosed with episodic migraine and prescribed a preventive, topiramate, titrated up to 50 milligrams (mg) twice a day for a month. This medication provided partial relief but was discontinued due to intolerable side effects that included tingling of extremities and loss of taste. She was subsequently switched to a CGRP antagonist, once a month intra-muscular fremanezumab, which afforded her significant relief of the headaches. However, with resolution of the episodic migraine, a new headache emerged. This was different. This new headache settled over the right temple and peri-orbital area. It was constant and described as an intense pressure that waxed and waned 6-10 times a month. These exacerbations lasted up to 6 hours a day and were not associated with photophobia or phonophobia, as with the previous headaches. However, she always suffered from a constant right peri-orbital headache that she graded 6 out of 10 on a visual analog scale. Of note, she denied ptosis, tearing, itching of the eye, tearing, aural fullness or pupillary changes. She occasionally suffered a runny nose, nasal congestion, a puffy eyelid and conjunctival injection during the headache. However, these latter symptoms were variable and not always consistent with the headache. She also denied any change in the frequency or quality of the menses, loss of libido or galactorrhea. She also denied any frontal bone bossing, loss of eyebrow hair or thickening of fingers or hands. Other than endometriosis and minor depression, she was healthy. Her medications included paroxetine and estrogen. Family history was negative for any primary headache disorder.

Her examination was significant for a blood pressure (BP) of 117/79 and a pulse of 59, with a height of 5 feet 5 inches with a weight of 184 pounds and a body-mass index (BMI) of 30.6. Her gait cadence and tandem walking was normal. Precordial auscultation revealed no cardiac murmurs and carotid auscultation no bruits.

Relevant examination especially cranial nerve examination was normal. Funduscopic examination revealed no papilledema. Ocular motility was full in all directions. No pupillary asymmetry was noted, specifically no ptosis or miosis. Accommodation was preserved. Visual fields were full to confrontation. No facial asymmetry was noted and no facial anesthesia. Palate elevation was symmetric with gag and tongue protrusion was midline. Shoulder shrug was symmetric. Power using Medical Research Council (MRC) grading was 5/5 throughout the upper and lower extremities. No dysmetria or intention tremor was noted. Deep tendon reflexes were lively and symmetric in the arms and legs. Sensory examination to touch, pressure, joint position sense was normal in the finger and toes.

**Figure 1:**
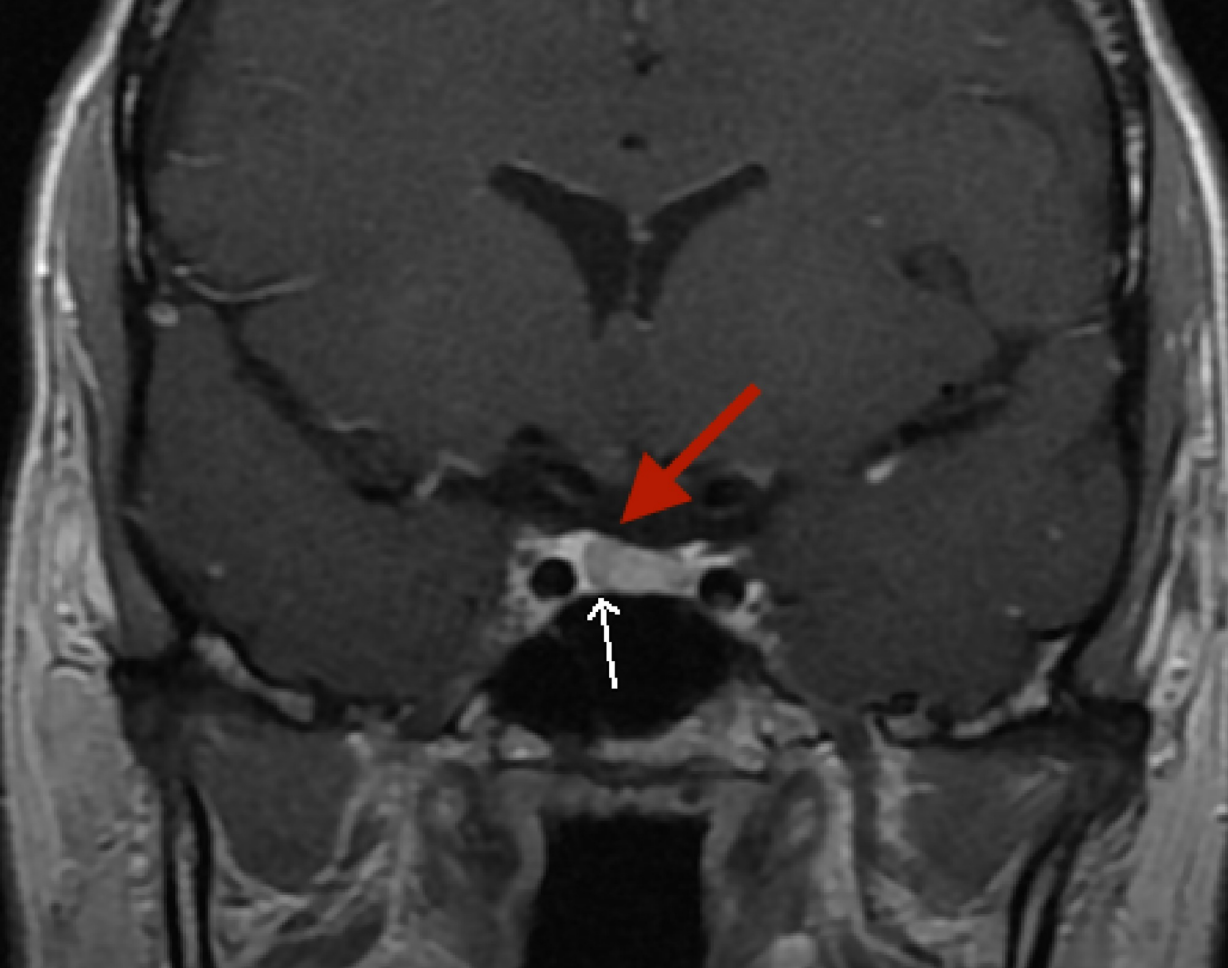
Gadolinium-enhanced T1-weighted coronal MRI: subtle right pituitary hypo-enhancement (white arrow) with elevation of diaphragmatic sella (red arrow) MRI: Magnetic Resonance Imaging

Due to persistence of the headaches, a nuclear magnetic resonance imaging (MRI) of the brain was obtained. A coronal gadolinium enhanced T1 weighted MRI view revealed a 5.2 millimeter (mm) right pituitary micro-adenoma. This is visible as a subtle hypointensity with an elevated diaphragmatic sella (Figure 1).

**Figure 2:**
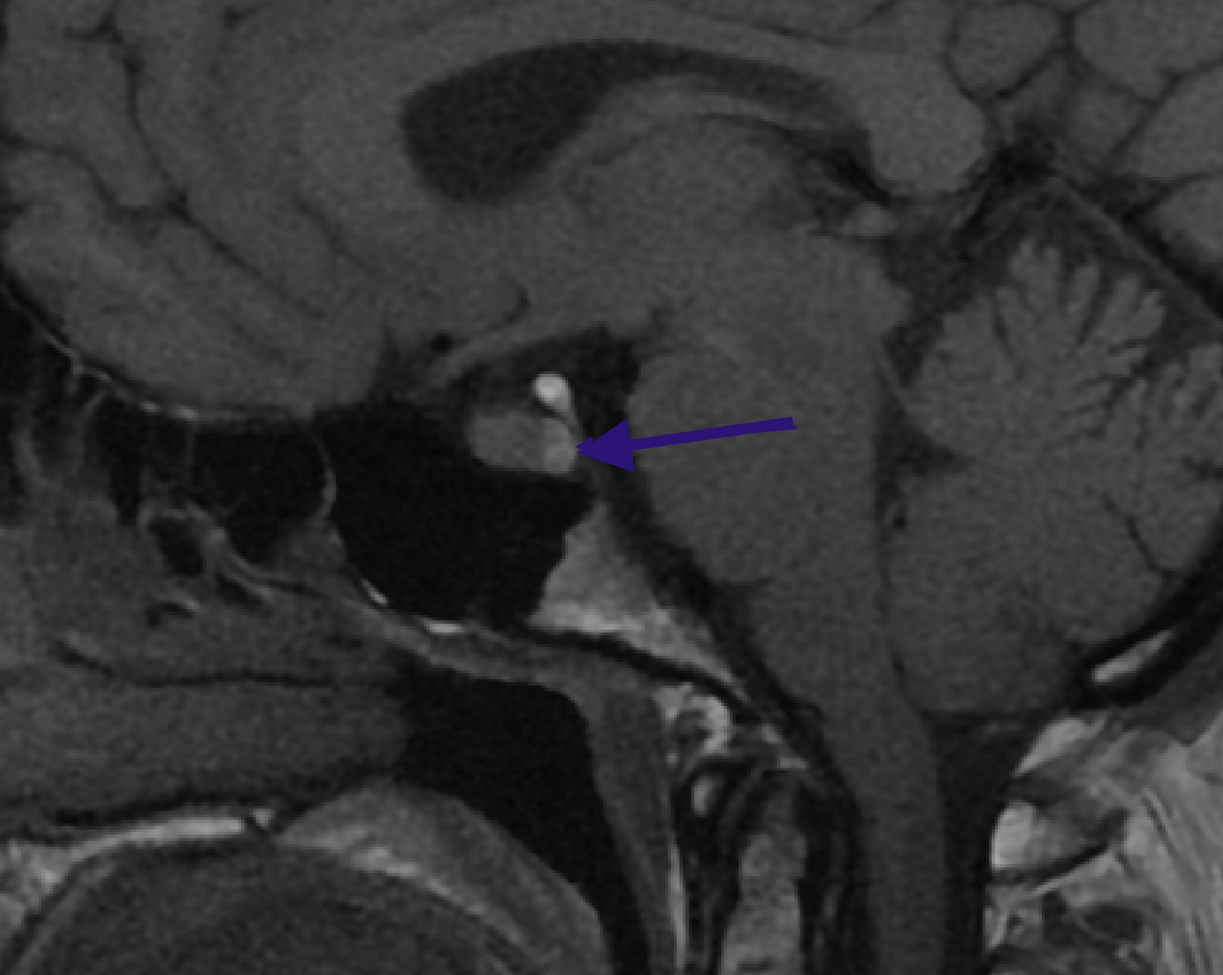
T1-weighted sagittal MRI: normal posterior pituitary hyperintense signal (blue arrow) MRI: Magnetic Resonance Imaging

A sagittal T1 weighted MRI view revealed the usual posterior pituitary high intensity signal (Figure 2).

A magnetic resonance angiography (MRA) did not reveal any intra-cranial cerebral aneurysm.

Serum thyroid stimulating hormone (TSH), serum follicle stimulating hormone (FSH), serum luteinizing hormone (LH), serum prolactin (PRL) and serum insulin growth factor (IGF) were within normal limits. The patient received indomethacin 25 mg three times daily for three days, which was then titrated up to 50 mg three times daily. After seven days of therapy, the patient's headaches improved significantly. However, side effects from indomethacin, including itching and dizziness were intolerable. Subsequently, the patient underwent a right occipital, supra-orbital nerve block and auriculo-temporal nerve block with partial relief of headaches. The patient is being monitored carefully to determine if she needs further nerve blocks or botulinum toxin injections.

## Discussion

The cranial dura mater of the middle cranial fossa is richly innervated by afferent nerve fibers from all three branches of the trigeminal nerve, which originate from the ipsilateral trigeminal ganglion, and by sympathetic fibers arising from the ipsilateral superior cervical ganglion. In addition, a sparse parasympathetic innervation arises from the ipsilateral sphenopalatine ganglion. The meningeal sensory neurons in the dura initially course alongside the meningeal arteries en route to their innervation, but the individual nerve ﬁbers exit the main bundle and travel some distance away from the artery before reaching their area of innervation and the majority of nerve endings are not in close proximity to arteries [6]. Activation of the trigeminovascular system within the cranial dura mater is known to be a cause of headaches [7].

The maxillary nerve (V2) originates from the trigeminal Gasserian ganglion which is located in Meckel’s cave, which is formed by the two layers of the dura mater. The Gasserian ganglion can be found near the apex of the petrous part of the temporal bone. There is no consent as to whether or not the maxillary nerve (V2) passes within the cavernous sinus or is embedded in the lateral wall of the cavernous sinus. After arising from the trigeminal ganglion, the maxillary nerve (V2) passes beneath the dura of the middle fossa, below where the medial and lateral walls of the cavernous sinus fuse, at the lower edge of the ophthalmic nerve (V1). The maxillary nerve exits the skull through the foramen rotundum and enters into the sphenopalatine fossa. Before exiting, it gives off several branches including the meningeal branches. It is a purely sensory nerve carrying both polymodal nociceptive mechanoreceptors and proprioceptive receptors [8].

A study of 84 patients with pituitary tumors who had headaches were studied. Forty-six percent presented with chronic migraine, 30% with episodic migraine, 5% with short lasting unilateral neuralgiform headache attacks with conjunctival injection and tearing (SUNCT), 4% with cluster headaches, 1% with hemicrania continua and 27% with primary stabbing headache. Seven per cent were unclassified. Twenty-one per cent had cavernous sinus invasion, which was present in two of three patients with cluster headaches. SUNCT headaches were only seen in patients with acromegaly and prolactinoma. Hypophysectomy improved headaches in 49% of patients. Somatostatin analogues improved acromegaly-associated headache in 64% of cases. Dopamine agonists improved headache in 25% of cases. Therefore a range of headache phenotypes can occur with pituitary tumors [9]. Furthermore, the trigeminal autonomic cephalgias (TAC) which comprise cluster headaches, SUNCT and HC are overrepresented in pituitary adenomas (10%), one hundred-fold over baseline, which should be 0.1% of the general population.

Many studies have looked at intra-sellar pressure ISP with pituitary adenomas [10-12].

The sella turcica is a rigid and inelastic space. The growth of a tumor in this fossa is likely to cause an increase of ISP. It is intuitive that a small increase of ISP may compress the anterior pituitary and disrupt endocrine function. It is known that the feeding portal veins have a low pressure. It is also unlikely that the ISP exceeds intracranial pressure (ICP). The ISP has been shown to be elevated in patients with pituitary tumors. The highest pressures were recorded in tumors with para-sellar invasion irrespective of the size and extent of invasion. There is also no correlation between the level of raised ISP and the tumors size. It is thought that the highest ISP pressures are found in macro-adenomas that have not broken through the confines of the pituitary fossa [10].

ISP was measured at trans-sphenoidal surgery in 107 patients with pituitary adenomas or intra-sellar cysts. ISP in patients with small micro-adenomas (<5 mm diameter) or a partial empty sella was 9-15 mmHg. Raised ISP (>15 mmHg) was found in 75% of cases. The highest pressures were recorded in tumors with para-sellar invasion, 28-32 mmHg, irrespective of size and extension. In non-invasive lesions there was no correlation between the level of raised ISP and tumor size. Hypopituitarism and stalk compression syndrome were both associated with higher ISP than patients with normal pituitary function [11].

Increased ISP can diminish perfusion pressure to the normal pituitary. Most patients with large adenomas have ISP that is higher than systemic venous pressure. Since the portal vessels are similar in structure to peripheral veins, it is reasonable to expect that even a minor elevation in ISP would diminish blood flow to the pituitary. It is also posited that compromised blood flow to the pituitary leads to ischemic necrosis and may lead to activation of autonomic fibers leading to pain. The most logical explanation is that an elevation of ISP leads to meningeal stretching and activation of nerve fiber endings. However, this may not hold true for a pituitary micro-adenoma [12].

The nerve supply of the pituitary body mirrors the arterial supply. The anterior lobe of the pituitary receives an extensive blood supply from a large number of minute vessels which radiate from the circle of Willis to the hypophyseal stalk, akin to the spokes of the hub of a wheel. The majority of these branches are from the anterior and posterior communicating arteries. The sympathetic nerves along the carotid plexus are continuous along the trifurcation of the carotid artery into the anterior cerebral, middle cerebral and posterior communicating arteries. A few fibers continue along the anterior and middle cerebral arteries for a short distance but the majority are found on the two communicating arteries which supply the pituitary. From the carotid plexus numerous branches are given off and pass along the blood vessels to the stalk of the pituitary, from which they penetrate into the anterior lobe of the pituitary [13]. As outlined in the next paragraph, the parasympathetic system supplies the intra-dural blood vessels, and these are implicated in the TACs.

**Figure 3:**
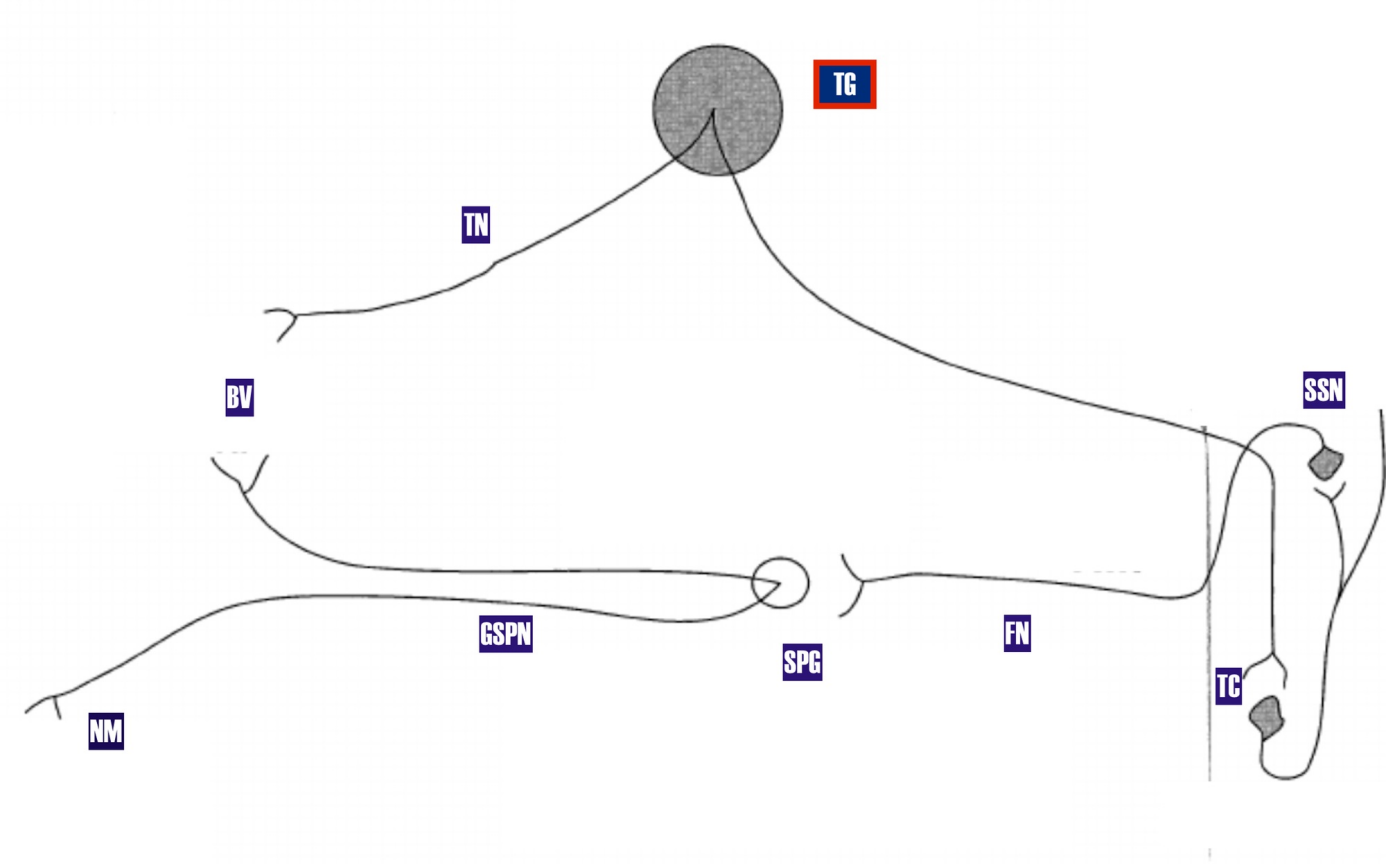
Pathway of trigemino-autonomic reflex: The superior salivatory nucleus is the integrator of the afferent arc (trigeminal nerve) and the efferent arc (facial nerve and SPG) SPG: Sphenopalatine ganglion; FN: Facial nerve; SSN: Superior salivatory nucleus; BV: Blood vessel; TN: Trigeminal nerve; TC: Trigeminal complex; NM: Nasal mucosa.

The TACs, of which HC is one type, the others being cluster headaches and SUNCT, are characterized by facilitation of the trigeminal autonomic reflex. This reflex has its afferent arc from the ophthalmic (V1) division of the trigeminal nerve. The neural integrator or central autonomic processor is the posterior hypothalamus and superior salivatory nucleus (SSN). The efferent arc from the SSN is via the seventh cranial nerve to the sphenopalatine ganglion and hence via the greater superficial petrosal nerve [14]. A cartoon of the trigeminal autonomic reflex arc is displayed below (Figure 3).

Indomethacin influences cerebral hemodynamics. Indomethacin lowers ICP, improves cerebral perfusion pressure, and leads to a dose-related reduction of the cerebral blood flow (CBF). Three mechanisms cause CBF reduction: 1) cyclo-oxygenase inhibition prevents the formation of vasodilating prostaglandins, 2) induction of hyperventilation leads to mild decreases in arterial carbon dioxide tension, and 3) vasoconstriction of cerebral blood vessels. Other cyclo-oxygenase inhibitors have no effect on CBF. Low doses of indomethacin have been shown to reduce CBF. The ICP lowering effects of indomethacin are thought to be best explained by vasoconstriction and a reduction of CBF. After intravenous administration of indomethacin, there is a significant increase in the arteriovenous oxygen difference without an increase in lactate formation, and hence precluding ischemia. Cerebral auto-regulation is preserved and there is a rebound increase in ICP after drug discontinuation [15-17].

Indomethacin is also known to inhibit the SSN and diminish activity in the trigemino-autonomic reflex [18]. Therefore, we have all the ingredients to explain the unilateral pain of HC in pituitary micro-adenomas. Mass effect by disrupting local tissue structure may perturb both the sympathetic and parasympathetic nerve supply, the latter activating the trigemino-autonomic reflex, which is known to lead to headaches [8]. A slight rise in ISP may lead to portal venous hypertension which may also activate the parasympathetic pathway. The sella turcica is a rigid box, and it is very conceivable that a slight rise in pressure may stretch the dura and activate the trigeminal nociceptive fibers. Last but not least, indomethacin which lowers ICP, reduces cerebral blood flow and inactivates the trigemino-autonomic reflex arc, is the most effective treatment for HC.

## Conclusions

Our case of HC associated with pituitary micro-adenoma has been described before. What we have done uniquely in this paper is to stitch together all the different facets of HC under one umbrella. We outline the nerve supply of the pituitary, flesh out the basics of the trigeminal autonomic reflex, discuss the dynamics of ISP and lay out the mechanisms of action of indomethacin: its effect on cerebral blood flow, ICP and the trigemino-autonomic reflex. These seemingly disparate concepts were combined under one edifice. The pathophysiology laid down in this article allows us to surmise that the association of pituitary micro-adenomas and HC is not spurious and is driven by well-defined hodology and physiological mechanisms.
